# Sequential Folliculitis, Paraspinal Myositis, and Septic Arthritis Due to Hematogenous Staphylococcus aureus Infection in a Patient Receiving Adalimumab Therapy

**DOI:** 10.7759/cureus.99143

**Published:** 2025-12-13

**Authors:** Ethan Burton, William Haller

**Affiliations:** 1 Osteopathic Medicine, Edward Via College of Osteopathic Medicine, Auburn, USA; 2 Orthopedics, Gadsden Regional Medical Center, Gadsden, USA

**Keywords:** adalimumab, bacteremia, hematogenous spread, paraspinal pyomyositis, staphylococcus aureus, tnf-alpha inhibitors

## Abstract

Skin infections are a well-recognized source of bacteremia and can serve as foci for hematogenous spread leading to sepsis, septic arthritis, or osteomyelitis. Immunosuppressive therapies, particularly tumor necrosis factor alpha (TNF-α) inhibitors such as adalimumab, predispose patients to severe infections and are associated with increased infectious morbidity and mortality. These agents impair innate immune signaling and may attenuate the clinical manifestations of infection. Specifically, TNF-α blockade disrupts downstream interleukin-1β (IL-1β) and interleukin-6 (IL-6) pathways that mediate leukocyte recruitment and the febrile response. Consequently, patients receiving TNF-α inhibitors may present with bacteremia or early sepsis in the absence of typical findings such as fever or leukocytosis, potentially delaying diagnosis and treatment.

We report a middle-aged Caucasian male patient receiving adalimumab therapy who initially presented to the emergency department with presumed paraspinal muscle strain and degenerative spondylosis. Magnetic resonance imaging (MRI) was interpreted as consistent with muscular strain and degenerative spondylosis. Subsequent orthopedic evaluation revealed a septic-appearing left knee, with synovial and blood cultures positive for methicillin-sensitive *Staphylococcus aureus *(MSSA). Further review of his history revealed that he had been treated for folliculitis over the occipital scalp one week prior to presentation, suggesting hematogenous dissemination from a cutaneous source.

Given the patient’s immunosuppressed state and atypical presentation, we conclude that bacteremia secondary to folliculitis resulted in occult paraspinal myositis mimicking a muscle strain, and ultimately progressed to septic arthritis. This case highlights the need for high clinical suspicion for hematogenous infection in patients receiving TNF-α inhibitors who present with musculoskeletal pain and minimal systemic findings.

## Introduction

*Staphylococcus aureus* (*S. aureus*) is the leading cause of both septic arthritis and pyomyositis, a bacterial infection of skeletal muscle [1,2]. Cutaneous infections such as folliculitis and furunculosis provide *S. aureus* entry portals necessary for hematogenous dissemination to deeper tissues in the body; specifically, local trauma-associated with vigorous use-and rich vascular supply are targets for seeding [1,3,4]. The use of immunosuppressive therapy, specifically adalimumab, a tumor necrosis factor alpha (TNF-α) inhibitor, is associated with increased incidence, morbidity, and mortality in the context of bacterial infections [5]. TNF-α inhibitors interfere with immune cell activation, proliferation, and inflammatory cytokine production-in the context of bacterial infection, this includes impaired macrophage activation and neutrophil recruitment [6]. Many of these cytokine-mediated processes underlie the clinical signs used in the quick Sequential (Sepsis-related) Organ Failure Assessment (qSOFA) and Sepsis-3 screening criteria [7]. Disruption of these mechanisms complicates early diagnosis and demonstrates the need for a lower threshold for work-up.

We present a case demonstrating the progression from *S. aureus* cutaneous infection to pyomyositis and finally septic arthritis in the setting of immunosuppression. This case illustrates the diagnostic challenges posed by attenuated inflammatory responses and the failure to recognize early warning signs, specifically tachycardia and relative monocytosis, suggestive of evolving infection. To our knowledge, few reports describe the sequential progression from folliculitis to pyomyositis and septic arthritis in a patient receiving TNF-α inhibitor therapy.

## Case presentation

A Caucasian male patient in his 50s with a history of psoriatic arthritis, degenerative joint disease, and lumbar spinal stenosis presented to the emergency department with worsening lower back pain for five days. The pain began after driving on a bumpy road, was aggravated by movement, and relieved by rest.

His past surgical history included a right total knee replacement. Aside from folliculitis over the posterior occiput treated empirically with oral amoxicillin a week prior, he had no other significant medical history. Current medications were adalimumab (Humira) subcutaneous injections every two weeks, cyclobenzaprine, meloxicam, meclizine, and rosuvastatin. His last adalimumab injection was administered one week prior to presentation.

On arrival, vital signs showed tachycardia (118 bpm) and mild hypertension (145/89 mm Hg), but the patient was afebrile and hemodynamically stable. The neurologic and musculoskeletal examinations were otherwise unremarkable. Physical examination demonstrated pain and a limited range of motion of the lower lumbar spine.

Abnormal laboratory findings are shown in Table 1. Compared to the reference range, there is mild monocytosis and lymphopenia. However, the values differ significantly from the patient’s baseline.

**Table 1:** Baseline Laboratory Values Contrasted With ER and Confirmed Infection Values Emergency room (ER) and confirmed infection values demonstrate lymphocytopenia, relative monocytosis, and neutrocytosis relative to baseline. These results are largely consistent with bacterial infection within the clinical context. Parameters reported include values outside the reference range or those showing significant variation from baseline measurements. All findings not listed were within normal limits. Baseline results were obtained during an emergency department visit one month prior, while the patient was receiving adalimumab therapy and was being evaluated for a noninfectious condition. Confirmed infection is defined as the date when the obtained blood culture results returned positive-two days after collection and three days after the initial ER presentation. Reference ranges are based on the hospital's reporting system. WBC: white blood cell

Parameter	Baseline	ER	Confirmed infection	Reference range
WBC (×10³/mcL)	5.58	7.01	10.1	4.8-10.8
Lymphocytes (%)	53	15.4	14.9	20-50
Lymphocytes (×10³/mcL)	2.96	1.09	1.5	1.0–4.8
Neutrophils (%)	37.3	72.9	73.9	44-77
Neutrophils (×10³/mcL)	2.08	5.16	7.46	2.7–7.7
Monocytes (%)	6.6	10.7	10.3	1.0-8.8
Monocytes (×10³/mcL)	0.37	0.76	1.04	0.01–0.6
Immature granulocytes (%)	0.2	0.6	0.4	0.0
Immature granulocytes (×10³/mcL)	0.01	0.04	0.04	0.0

**Figure 1:**
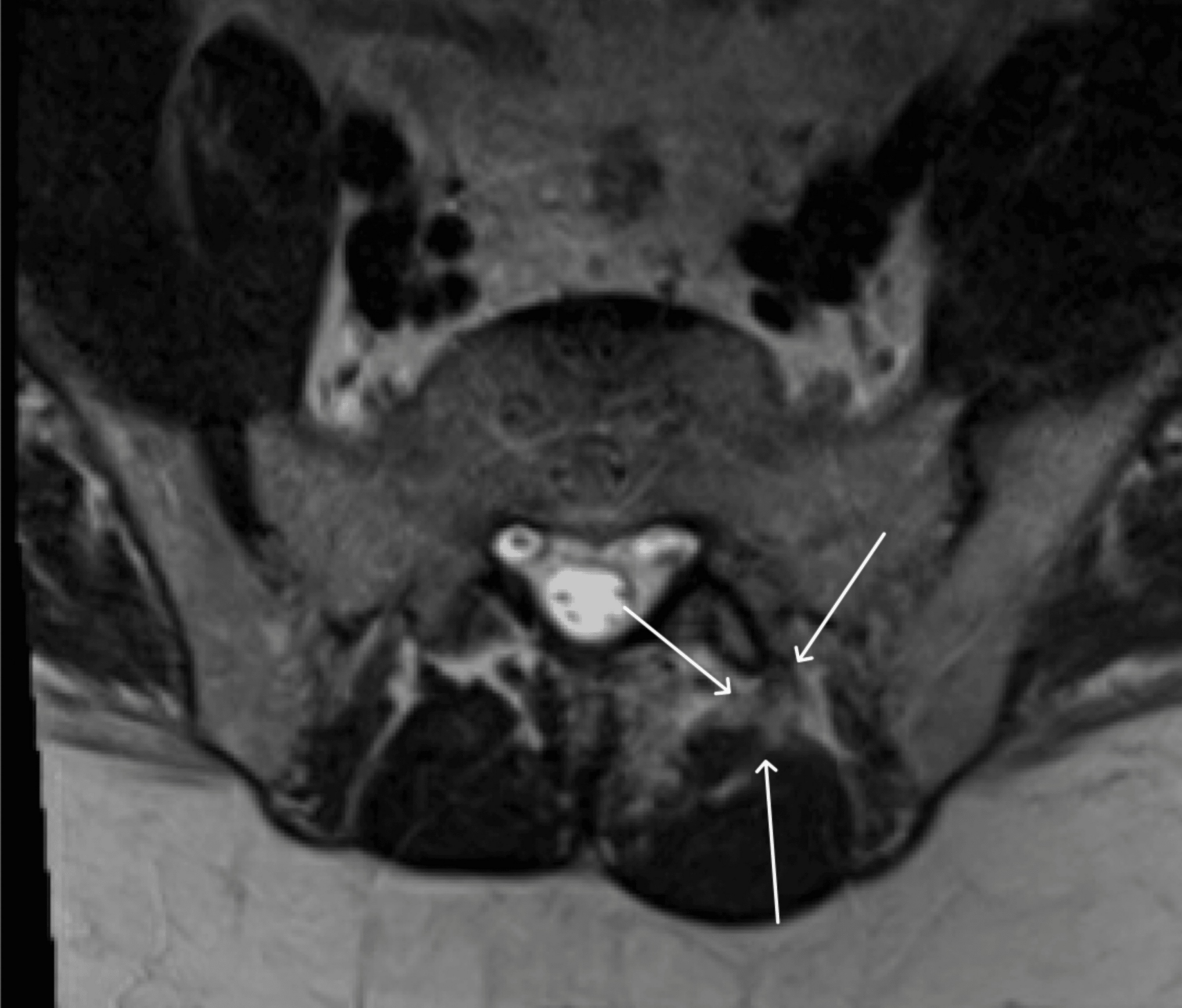
T2-Weighted MRI With Contrast in the Sacral Plane The MRI was interpreted as left paraspinal muscle edema extending from L2 to S2, largely consistent with pyomyositis in the clinical context. MRI: magnetic resonance imaging

Magnetic resonance imaging (MRI) of the lumbar spine demonstrated a left paraspinal fluid collection extending from L2 to S2, interpreted as consistent with a paraspinal muscle strain (Figure 1). The patient was discharged with outpatient orthopedic follow-up.

Two days later, he presented to the orthopedic clinic with new-onset left knee pain, swelling, and erythema, reporting complete resolution of his back pain. On examination, the left knee was warm, swollen, and erythematous, and his temperature was 100.4°F (38°C).

Arthrocentesis of the left knee yielded serosanguineous fluid with mucinous characteristics, showing 33,000 white blood cells (WBCs) with 92% polymorphonuclear neutrophils (PMNs). The fluid was sent for culture. The patient underwent arthroscopic irrigation and debridement, which revealed degenerative joint changes and serosanguineous effusion. The joint was irrigated with 3 L of normal saline and 1 L of hypochlorous acid solution.

Culture results from both the knee aspirate and intraoperative specimens grew methicillin-susceptible *S. aureus* (MSSA), confirming *S. aureus* septic arthritis. Empiric intravenous vancomycin and cefazolin were administered before and immediately after surgery. The patient initially improved, with resolution of fever and pain. The patient was transitioned to oral linezolid 600 mg every 12 hours for 14 days upon discharge and instructed to stop adalimumab therapy until completion of antibiotic therapy.

The patient was readmitted one day after discharge with fever (101.4°F), malaise, and tachycardia (114 bpm). Blood cultures obtained at readmission also grew MSSA. Intravenous cefazolin was reinitiated and continued until blood cultures remained negative for 48 hours. The patient’s symptoms resolved, and blood cultures were negative four days after readmission. Repeat MRI was deemed unnecessary given his clinical improvement and clearance of bacteremia.

Infectious disease consultation concluded that the paraspinal edema seen on the initial MRI was suggestive of early hematogenous seeding of *S. aureus* and that the absence of abscess formation reflected the patient’s immunosuppressed state and impaired inflammatory response due to adalimumab therapy. Based on the clinical course, imaging, and microbiology, the final diagnosis was MSSA septic arthritis of the left knee, preceded by probable transient paraspinal pyomyositis and bacteremia in the setting of adalimumab-induced immunosuppression.

## Discussion

TNF-ɑ inhibitors interfere with cytokine-mediated processes that underlie the clinical signs used in qSOFA and Sepsis-3, potentially delaying diagnosis [7-9]. TNF-ɑ inhibitors inhibit neutrophil and monocyte activation, secondarily decreasing interleukin-1 beta (IL-1β), interleukin-6 (IL-6), and interleukin-8 (IL-8) secretion [6]. This blunted inflammatory response may prevent bacterial clearance and delay the onset of systemic symptoms, potentially explaining why the progression of fever and leukocytosis was delayed.

As shown in Table 1, the patient’s laboratory findings were only mildly abnormal, including lymphocytopenia, relative monocytosis, and neutrocytosis relative to baseline. Although these values were near or within the reference range, they represented a significant deviation from the patient’s baseline, suggesting a blunted yet evolving inflammatory response consistent with TNF-ɑ inhibition and largely consistent with bacterial infection. Although the observed elevations were quantitatively mild, the relative shift from baseline indicated early immune activation. In the context of TNF-α blockade, such subtle laboratory changes may represent a blunted systemic inflammatory response, masking the severity of underlying infection.

TNF-ɑ inhibitors suppress key cytokines required for macrophage activation and abscess formation [10]. *S. aureus* has the potential to migrate from cutaneous infections and form abscesses in deeper tissues [11,12]. In this case, the paraspinal edema observed on MRI likely represented a failed or partially organized abscess rather than simple inflammation. This interpretation aligns with the known role of TNF-α and IL-1β in abscess wall formation and containment of infection [10,13]. Suppression of these cytokines by TNF-α inhibition may have impaired abscess maturation, allowing bacterial persistence and subsequent hematogenous spread. However, this interpretation is limited by the absence of follow-up imaging to confirm abscess evolution, though the patient’s clinical course and positive blood cultures strongly support hematogenous dissemination secondary to impaired abscess formation.

Staphylococci adhere to the skin, invade into the deeper dermis, and colonize distant tissues through a number of virulence factors. Adherence to the skin is achieved with clumping factors (ClfA, ClfB), fibronectin-binding proteins (FnBPs), and collagen adhesin (Cna), while penetration into deeper layers of the dermis is accomplished with the enzyme hyaluronidase [14,15]. This deeper penetration into the dermis gives bacteria access to blood and lymphatic vessels that can be used for migration to distant parts of the body. Thus, highly vascular structures like muscle and synovium of the joints are frequently colonized. Colonization of distant tissue-particularly muscles, joints, and bone-is mediated through biofilm formation. Adhesins and polysaccharide adhesins mediate the formation of biofilms [16]. In healthy individuals, TNF-α is a key mediator of abscess formation in response to *S. aureus* colonization of muscle or soft tissue, including pyomyositis and folliculitis [13].

Pyomyositis occurs more frequently in tropical regions, and the causative pathogen is primarily *S. aureus*. There have been multiple case reports of individuals developing pyomyositis following *S. aureus* skin colonization [17,18]. While there are studies showing increased incidence of bacterial infections with the use of TNF-ɑ inhibitors, there are few reports that detail a case of folliculitis preceding the development of suspected pyomyositis and later septic arthritis [1].

TNF-ɑ inhibitors suppress multiple aspects of the immune system’s response to infection and are considered a form of functional immunosuppression. Consequently, a higher degree of scrutiny should be exercised when evaluating patients with mild laboratory or vital sign abnormalities, even in the absence of fever. Such findings may represent a blunted yet active immune response to an underlying pathogen. Moreover, *S. aureus* skin infections pose a genuine risk for transient bacteremia, which should be considered when assessing patients with discordant clinical and laboratory findings. In afebrile patients, heart rates of 100-125 beats per minute (BPM) have been associated with a 50%-75% probability of sepsis [7], demonstrating that the patient’s initial heart rate of 118 BPM was likely correlated to early signs of infection. Previous studies have shown that patients receiving TNF-ɑ inhibitors are at increased risk for invasive bacterial infections, including *S. aureus* bacteremia and soft tissue infections [5,19].

## Conclusions

This case highlights the diagnostic complexity of bacterial infections in patients receiving TNF-α inhibitors, where immunosuppression may blunt the expected inflammatory response and delay recognition of systemic infection. The progression from folliculitis to probable paraspinal pyomyositis and ultimately septic arthritis illustrates the potential for *S. aureus* to disseminate hematogenously even when early findings appear mild or self-limited. Clinicians should maintain a high index of suspicion for deep or systemic infection in immunosuppressed patients presenting with subtle laboratory or imaging abnormalities. Early recognition and treatment are essential to prevent progression to invasive disease.
